# Supplementary material for: Neurexins regulate presynaptic GABAB-receptors at central synapses
Source: Nat Commun. 2021 Apr 22;12:2380. doi: 10.1038/s41467-021-22753-5 (PMC8062527; doi:10.1038/s41467-021-22753-5)

Supplementary information

Neurexins regulate presynaptic $GABA_B$-receptors at central synapses

Fujun Luo, Alessandra Sclip, Sean Merrill, and Thomas C. Südhof

Supplementary Fig.1 Supporting information related to to Figs. 1-2. The effect of SKF on inhibition of transmitter release is reversible (a) and specifically blocked by $GABA_B$-receptor antagonist CGP 55845 (b).

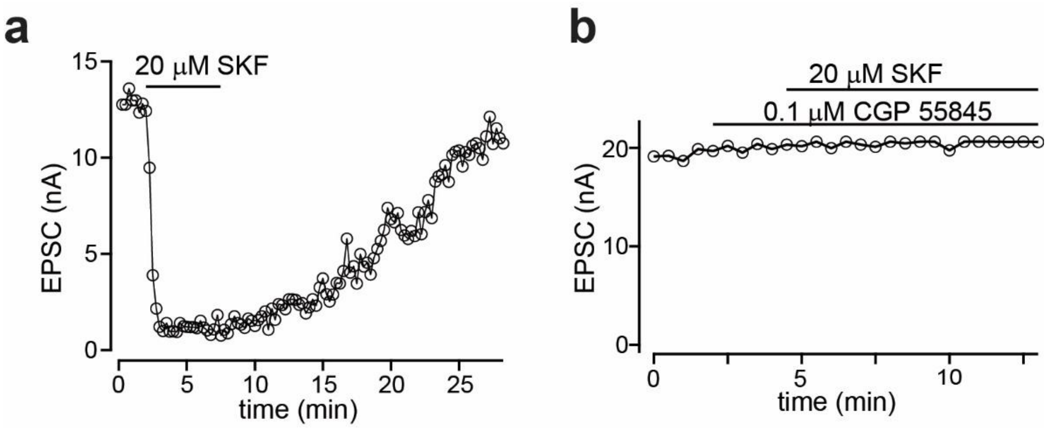

Supplementary Fig.2 Supporting information related to Figs.1-2. SKF effect on sEPSCs at the calyx of Held synapse. a Representative traces of sEPSCs before and after SKF recorded in acute slices from littermate control and neurexin123 TKO mice. **b** Summary graphs of sEPSC amplitude and frequency. For amplitude, P = 0.6295 (Ctrl.-SKF vs. Ctrl.+SKF), P = 0.99 (TKO-SKF vs. TKO+SKF), paired two-sided t-test. P = 0.1274 (Ctrl.-SKF vs. TKO-SKF), unpaired two-sided t-test. For frequency, P = 0.3081 (Ctrl.-SKF vs. Ctrl.+SKF), P = 0.5344 (TKO-SKF vs. TKO+SKF), paired two-sided t-test. P = 0.6159 (Ctrl.-SKF vs. TKO-SKF), unpaired two-sided t-test. **c** Summary graphs of sEPSC rise time and decay time constant. For rise time, P = 0.4573 (Ctrl.-SKF vs. Ctrl.+SKF), P = 0.0917 (TKO-SKF vs. TKO+SKF), paired two-sided t-test. P = 0.7931(Ctrl.-SKF vs. TKO-SKF), unpaired two-sided t-test. For decay τ, P = 0.8043 (Ctrl.-SKF vs. Ctrl.+SKF), P = 0.3534 (TKO-SKF vs. TKO+SKF), paired two-sided t-test. P = 0.389 (Ctrl.-SKF vs. TKO-SKF), unpaired two-sided t-test. Data are means ± SEM. Number of cells analyzed are indicated in the bars (**b**, **c**); No statistical differences was found by Student's t test. Source data are provided as a Source Data file.

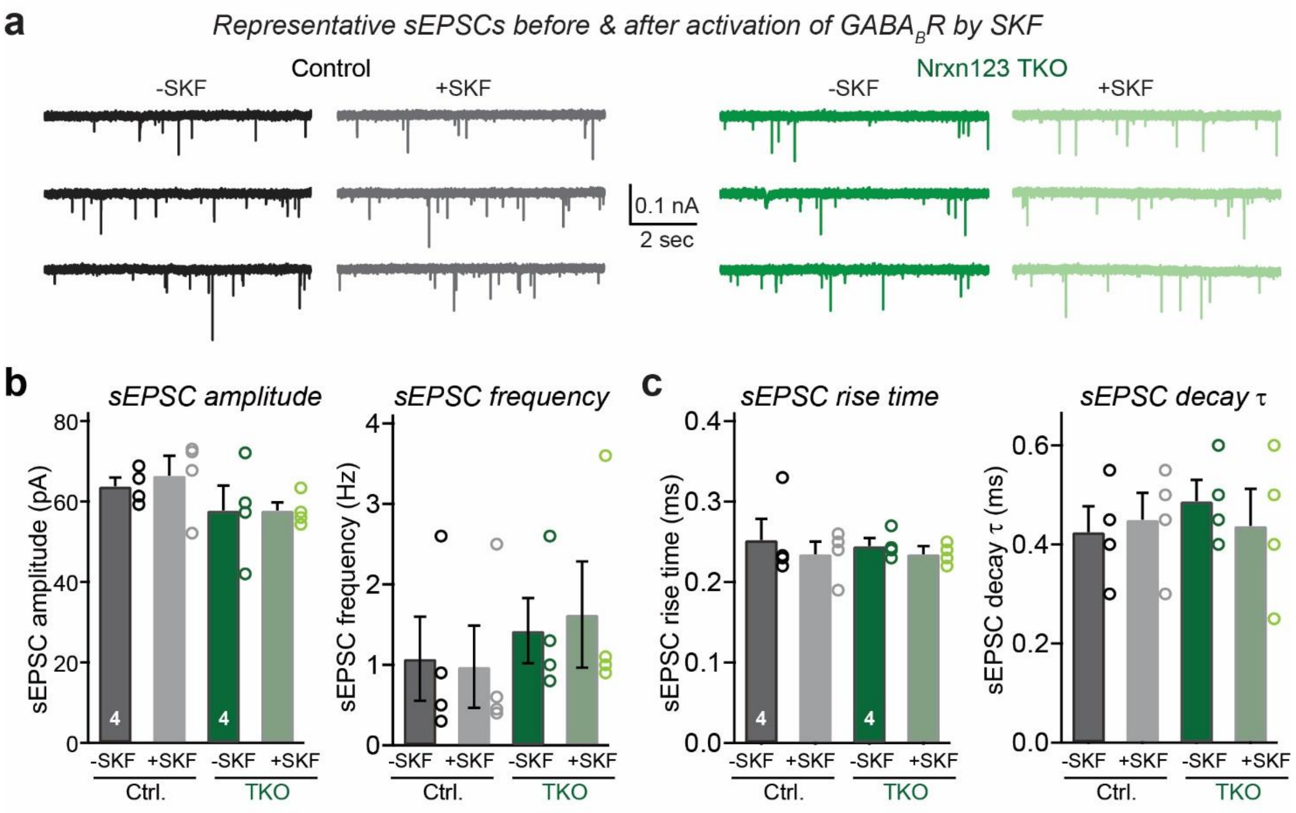

Supplementary Fig.3 Supporting information related to Figs. 4.

a Summary of the volume and particle count of $GABA_{B2}$ clusters. P = 0.045 (left), P = 0.0589 (right), unpaired two-sided t-test. **b** Summary of the volume and particle count of Homer1 clusters. P = 0.8844 (left), P = 0.9978 (right), unpaired two-sided t-test. **c, d** Same as (**a, b**), except for Homer1 (magenta) and $GABA_B$-receptor subunit 1 ($GABA_{B1}$, yellow). P = 0.9686 (left), P = 0.7697 (right), unpaired two-sided t-test in (**c**). P = 0.8777(left), P = 0.8959 (right), unpaired two-sided t-test in (**d**). Data are means ± SEM. Number of sections/animals for immunostaining are indicated in the bars. Statistical differences were assessed by Student's t test. (*P < 0.05). Source data are provided as a Source Data file.

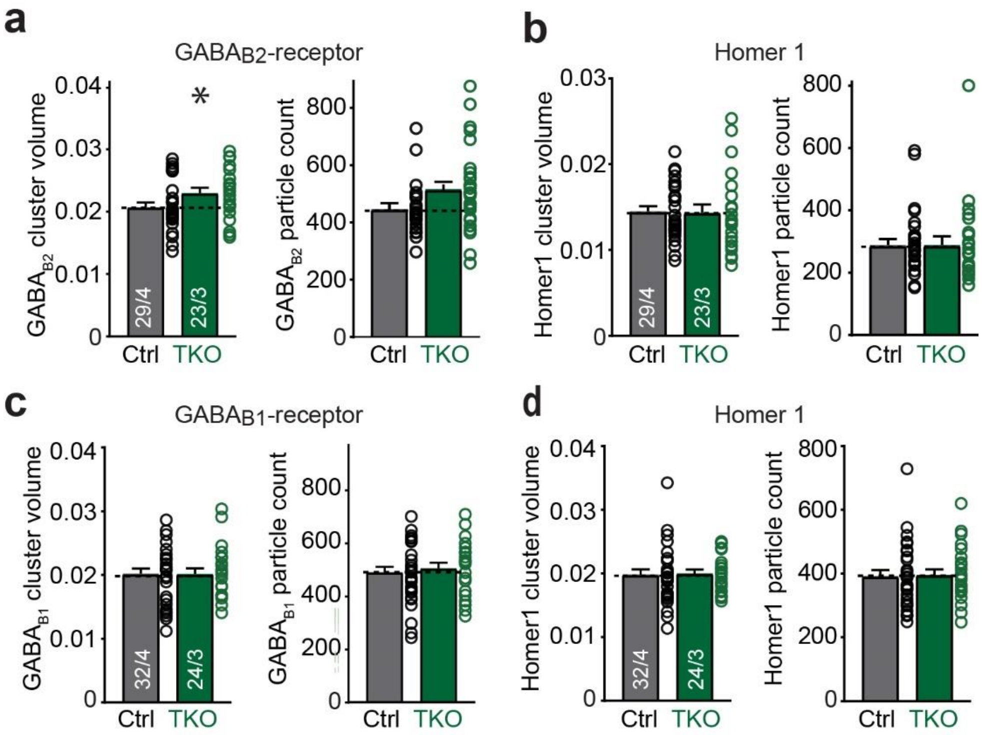

Supplementary Fig.4 Supporting information related to Figs. 5-6. SKF effect on sEPSCs and sIPSCs in hippocampal CA3 pyramidal cells. a Representative traces of sEPSCs before and after SKF for control (ΔCre) and Nrxn123 TKO synapses (Cre). **b** Summary graphs of sEPSC amplitude and frequency. For amplitude: P = 0.999 (ΔCre-SKF vs. ΔCre+SKF), P = 0.999 (Cre-SKF vs. Cre+SKF), paired two-sided t-test. P = 0.999 (ΔCre-SKF vs. Cre-SKF), unpaired two-sided t-test. For frequency: P = 0.999 (ΔCre-SKF vs. ΔCre+SKF), P = 0.999 (Cre-SKF vs. Cre+SKF), paired two-sided t-test. P = 0.999 (ΔCre-SKF vs. Cre-SKF), unpaired two-sided t-test. **c** Representative traces of sIPSCs before and after addition of $GABA_B$-receptor agonist SKF recorded in acute hippocampus slices from $PVCre^+$ mice (control) or $PVCre^+$/Nrxn123 cKO mice (TKO) injected with AAV-DIO-Chief-TdTomato. **d** Summary graphs of sIPSC amplitude and frequency. For amplitude: P = 0.0008 (Ctrl.-SKF vs. Ctrl.+SKF), P = 0.999 (TKO-SKF vs. TKO+SKF), paired two-sided t-test. P = 0.0003 (Ctrl.-SKF vs. TKO-SKF), unpaired two-sided t-test. For frequency: P = 0.0003 (Ctrl.-SKF vs. Ctrl.+SKF), P = 0.1488 (TKO-SKF vs. TKO+SKF), paired two-sided t-test. P = 0.1012 (Ctrl.-SKF vs. TKO-SKF), unpaired two-sided t-test. Data are means ± SEM. Number of cells (from at least three mice per group) analyzed are indicated in the bars (**b**, **d**); Statistical differences were assessed by Student's t test. (***P < 0.001). Source data are provided as a Source Data file.

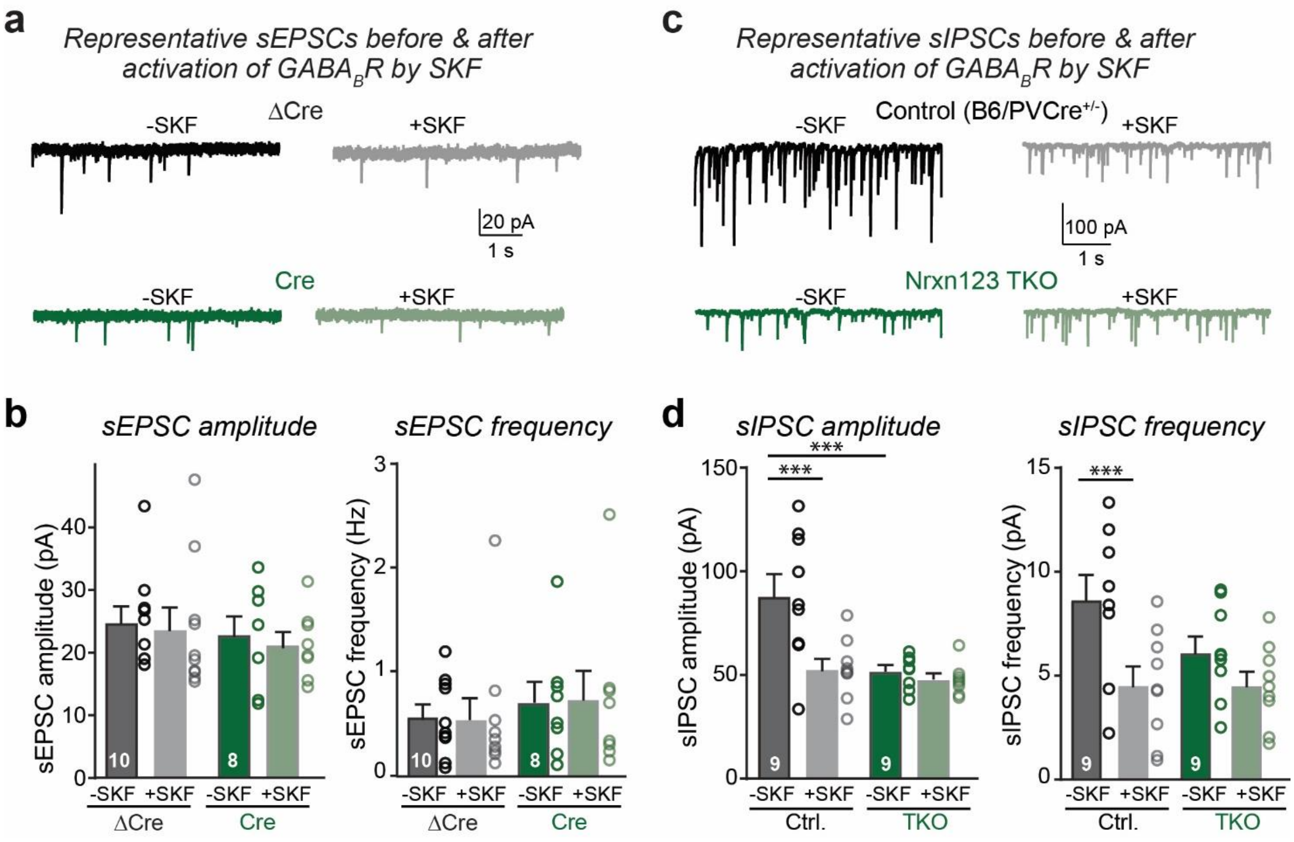

Supplementary Fig.5 Supporting information related to Fig.6. IPSCs recorded from CA1 pyramidal cells can be blocked by application of $GABA_A$-receptor antagonist PTX, confirming the GABAergic synapse formed between PV^+-interneuron and CA1 pyramidal cells.

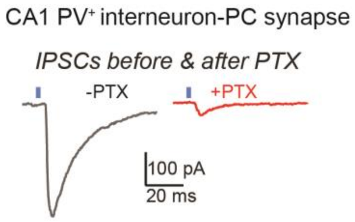

Supplementary Fig.6 Supporting information related to Fig.7. IPSCs recorded from cereblIar Purkinje cells can be blocked by application of $GABA_A$-receptor antagonist PTX, confirming the GABAergic synapse formed between cereblIar basket cells and Purkinje cells.

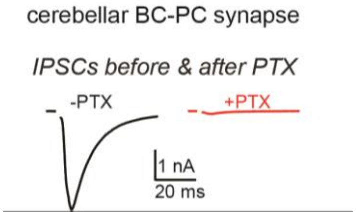

Supplementary Fig.7 Supporting information related to Fig.7. Effect of $GABA_B$-receptors on sIPSCs in cerebellar Purkinje cells. a Representative traces of sIPSCs before and after SKF recorded in acute slices from littermate control and neurexin123 TKO mice. **b** Summary graphs of sIPSC amplitude and frequency. For amplitude, P = 0.97 (Ctrl.-SKF vs. Ctrl.+SKF), P = 0.8818 (TKO-SKF vs. TKO+SKF), paired two-sided t-test. P = 0.1717(Ctrl.-SKF vs. TKO-SKF), unpaired two-sided t-test. For frequency, P = 0.1068 (Ctrl.-SKF vs. Ctrl.+SKF), P = 0.0727 (TKO-SKF vs. TKO+SKF), paired two-sided t-test. P = 0.0068 (Ctrl.-SKF vs. TKO-SKF), unpaired two-sided t-test. **c** Summary graphs of sIPSC rise time and decay τ. For rise time, P = 0.4086 (Ctrl.-SKF vs. Ctrl.+SKF), P = 0.6941 (TKO-SKF vs. TKO+SKF), paired two-sided t-test. P = 0.228 (Ctrl.-SKF vs. TKO-SKF), unpaired two-sided t-test. For decay τ, P = 0.0698 (Ctrl.-SKF vs. Ctrl.+SKF), P = 0.8839 (TKO-SKF vs. TKO+SKF), paired two-sided t-test. P = 0.3449 (Ctrl.-SKF vs. TKO-SKF), unpaired two-sided t-test. Data are means ± SEM. Number of cells (from at least three mice per group) analyzed are indicated in the bars (**b**, **c**); Statistical differences were assessed by Student's t test. (**P < 0.01). Source data are provided as a Source Data file.

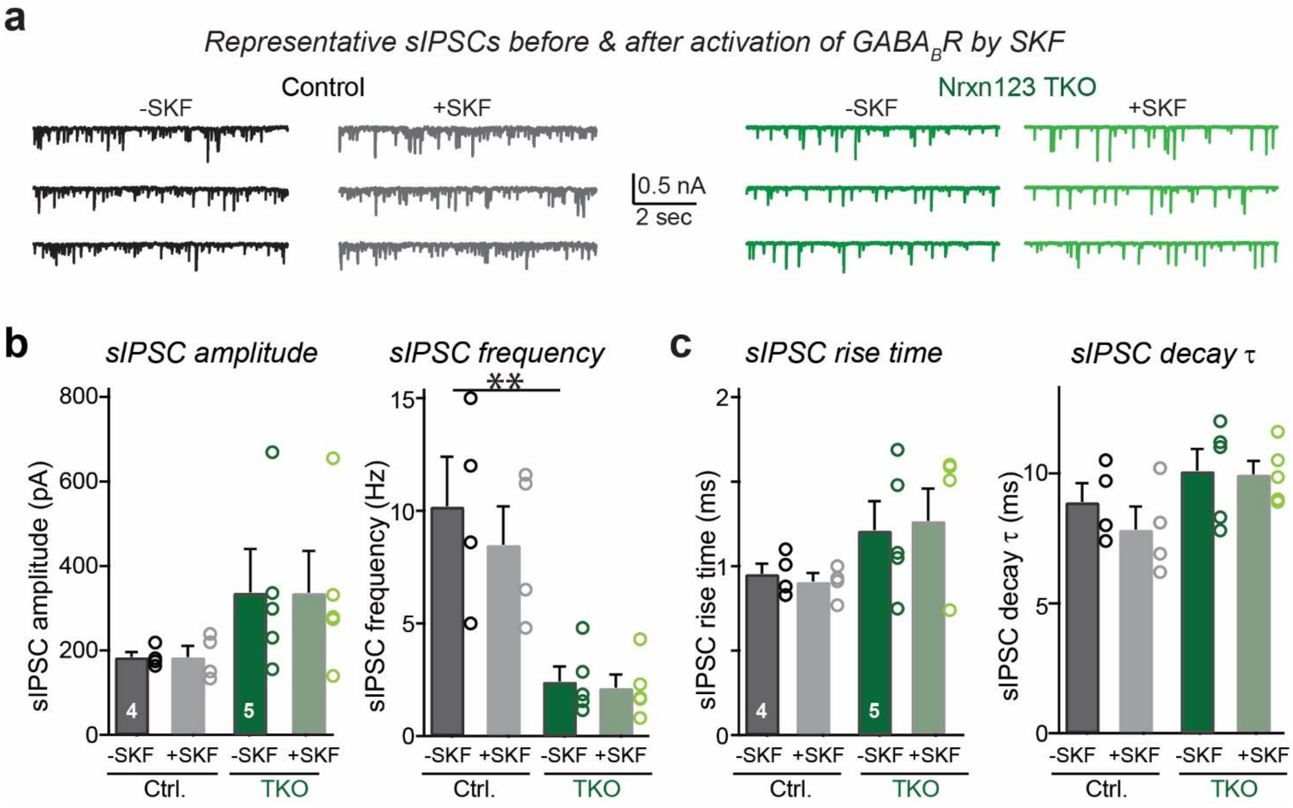

Supplement: Supplementary file 1 — Supplementary Information [file 41467_2021_22753_MOESM1_ESM.pdf]
