# Supplementary material for: Neurexins regulate presynaptic GABAB-receptors at central synapses
Source: Nat Commun. 2021 Apr 22;12:2380. doi: 10.1038/s41467-021-22753-5 (PMC8062527; doi:10.1038/s41467-021-22753-5)
Supplement: Supplementary file 3 — Reporting Summary [file 41467_2021_22753_MOESM3_ESM.pdf]

nature research

Corresponding author(s): Fujun Luo

Last updated by author(s): 2/28/2021

Reporting Summary

Nature Research wishes to improve the reproducibility of the work that we publish. This form provides structure for consistency and transparency in reporting. For further information on Nature Research policies, see Authors & Referees and the Editorial Policy Checklist .

Statistics

For all statistical analyses, confirm that the following items are present in the figure legend, table legend, main text, or Methods section.

n/a	Confirmed	
☐	☒	The exact sample size (*n*) for each experimental group/condition, given as a discrete number and unit of measurement
☐	☒	A statement on whether measurements were taken from distinct samples or whether the same sample was measured repeatedly
☐	☒	The statistical test(s) used AND whether they are one- or two-sided<br>*Only common tests should be described solely by name; describe more complex techniques in the Methods section.*
☐	☒	A description of all covariates tested
☒	☐	A description of any assumptions or corrections, such as tests of normality and adjustment for multiple comparisons
☐	☒	A full description of the statistical parameters including central tendency (e.g. means) or other basic estimates (e.g. regression coefficient) AND variation (e.g. standard deviation) or associated estimates of uncertainty (e.g. confidence intervals)
☐	☒	For null hypothesis testing, the test statistic (e.g. *F*, *t*, *r*) with confidence intervals, effect sizes, degrees of freedom and *P* value noted<br>*Give P values as exact values whenever suitable.*
☒	☐	For Bayesian analysis, information on the choice of priors and Markov chain Monte Carlo settings
☒	☐	For hierarchical and complex designs, identification of the appropriate level for tests and full reporting of outcomes
☐	☒	Estimates of effect sizes (e.g. Cohen's *d*, Pearson's *r*), indicating how they were calculated

Our web collection on statistics for biologists contains articles on many of the points above.

Software and code

Policy information about availability of computer code

Data collection	For patch clamp recording at the calyx of Held and the cerebellum, PatchMaster v2x73 (Heka, Lambrecht, Germany) was used. For patch recording at the hippocampus, Clampex 10.4 (Molecular Device, USA) was used. Confocal imaging was performed using Nikon A1RSi confocal microscope. dSTORM imaging was perfomred on Vutara SR 352 (Bruker Nanosurfaces, Inc., Madison, WI)
Data analysis	Igor Pro 6.0 was used for analysis of all electrophysiological data. Confocal imaging was analyzed in NIS-Elements Advanced Research software (Nikon). dSTORM imaging data was analyzed by Vutara SRX software (version 6.04)

For manuscripts utilizing custom algorithms or software that are central to the research but not yet described in published literature, software must be made available to editors/reviewers. We strongly encourage code deposition in a community repository (e.g. GitHub). See the Nature Research guidelines for submitting code & software for further information.

Data

Policy information about availability of data

All manuscripts must include a data availability statement. This statement should provide the following information, where applicable:

- Accession codes, unique identifiers, or web links for publicly available datasets
- A list of figures that have associated raw data
- A description of any restrictions on data availability

All relevant data supporting the findings of this study are available from the corresponding authors upon reasonable request.

Field-specific reporting

Please select the one below that is the best fit for your research. If you are not sure, read the appropriate sections before making your selection.

Life sciences study design

All studies must disclose on these points even when the disclosure is negative.

Sample size	No statistical methods were used to predetermine sample size, but the sample sizes were based on those in previously published studies (Chen et al., Neuron, 2017; Luo et al., EMBO J, 2020).
Data exclusions	No data were excluded from the analyses.
Replication	All experiments were successfully replicated from at least three animals per group.
Randomization	Mice were randomly assigned to groups for each experiment.
Blinding	All experiments were performed blindly by the experimenters without knowledge of the mouse genotypes.

Reporting for specific materials, systems and methods

We require information from authors about some types of materials, experimental systems and methods used in many studies. Here, indicate whether each material, system or method listed is relevant to your study. If you are not sure if a list item applies to your research, read the appropriate section before selecting a response.

Materials & experimental systems

n/a	Involved in the study	
☐	☒	Antibodies
☐	☒	Eukaryotic cell lines
☒	☐	Palaeontology
☐	☒	Animals and other organisms
☒	☐	Human research participants
☒	☐	Clinical data

Methods

n/a	Involved in the study	
☒	☐	ChIP-seq
☒	☐	Flow cytometry
☒	☐	MRI-based neuroimaging

Antibodies

Antibodies used

For immunohistochemistry and dSTORM imaging, the following antibodies were used:

VGluT1 (guinea pig, polyclonal, 1:1000, Millipore, Cat#: AB5905; RRID: AB_2301751)
GABAB R1 (mouse, monoclonal, 1:500-1000, NeuroMab Cat#: 75-183)
GABAB R2 (mouse, monoclonal, 1:500-1000, NeuroMab Cat#: 75-125)
Homer1 (rabbit, polyclonal, 1:1000, Millipore, Cat#: ABN37)
Secondary antibodies were Alexa Fluor conjugates (1:500-3000; Invitrogen; anti guinea pig Alexa 488 Cat# A11073, anti mouse Alexa 546 Cat# A10036, anti rabbit Alexa 647 Cat#21245) or CF568 (1:3000, Biotium, Cat#20802).

Validation

The vGlut1 antibody was used and validated in Refs 33, 34. The Homer1 antibody was validated on mouse brain tissue lysates by western blot as specified by the vendor. GABAB R1 and GABAB R2 antibodies were validated by Western blot on brain membrane fractions from KO mice and tested by immunocytochemistry as specified by the vendor.

Eukaryotic cell lines

Policy information about cell lines

Cell line source(s)	HEK 293T were directly purchased from ATCC
Authentication	HEK 293T were directly purchased from ATCC. The cell line was not authenticated (other than by morphology and passage).
Mycoplasma contamination	Cell lines were tested negative for mycoplasma contamination using the fluorochrome Hoechst DNA stain and the direct culture method.
Commonly misidentified lines (See ICLAC register)	None of the cell lines used is listed as commonly misidentified.

Animals and other organisms

Policy information about studies involving animals; ARRIVE guidelines recommended for reporting animal research

Laboratory animals	pV-Cre/Nrxn123 cKO mice at age P12-14 were used for the calyx of Held study; pV-Cre/Nrxn123 cKO mice and pV-Cre mice at age P35-42 were used for recording in hippocampus; pV-Cre/Nrxn123 cKO mice at age P35-42 were used for recording in cerebellum. Both male and female mice were used. Mice were housed at room temperature and 40-60% humidity on a light-dark cycle (07:00 to 19:00, light).
Wild animals	The study did not involve wild animals.
Field-collected samples	The study did not involve field-collected samples.
Ethics oversight	All experiments were approved by the Institutional Animal Care and Use Committee at Stanford University.

Note that full information on the approval of the study protocol must also be provided in the manuscript.
